# Closing the Yield Gap for Cannabis: A Meta-Analysis of Factors Determining Cannabis Yield

**DOI:** 10.3389/fpls.2019.00495

**Published:** 2019-04-24

**Authors:** Rachel Backer, Timothy Schwinghamer, Phillip Rosenbaum, Vincent McCarty, Samuel Eichhorn Bilodeau, Dongmei Lyu, Md Bulbul Ahmed, George Robinson, Mark Lefsrud, Olivia Wilkins, Donald L. Smith

**Affiliations:** ^1^Crop Physiology Laboratory, Department of Plant Science, McGill University, Sainte-Anne-de-Bellevue, QC, Canada; ^2^Plant Systems Biology Laboratory, Department of Plant Science, McGill University, Sainte-Anne-de-Bellevue, QC, Canada; ^3^Biomass Production Laboratory, Department of Bioresource Engineering, McGill University, Sainte-Anne-de-Bellevue, QC, Canada; ^4^Ravenquest Biomed, Inc., Vancouver, BC, Canada

**Keywords:** cannabis, genomics, transcriptomics, chemotype, yield gap, light emitting diodes, PGPR, GWAS

## Abstract

Until recently, the commercial production of *Cannabis sativa* was restricted to varieties that yielded high-quality fiber while producing low levels of the psychoactive cannabinoid tetrahydrocannabinol (THC). In the last few years, a number of jurisdictions have legalized the production of medical and/or recreational cannabis with higher levels of THC, and other jurisdictions seem poised to follow suit. Consequently, demand for industrial-scale production of high yield cannabis with consistent cannabinoid profiles is expected to increase. In this paper we highlight that currently, projected annual production of cannabis is based largely on facility size, not yield per square meter. This meta-analysis of cannabis yields reported in scientific literature aimed to identify the main factors contributing to cannabis yield per plant, per square meter, and per W of lighting electricity. In line with previous research we found that variety, plant density, light intensity and fertilization influence cannabis yield and cannabinoid content; we also identified pot size, light type and duration of the flowering period as predictors of yield and THC accumulation. We provide insight into the critical role of light intensity, quality, and photoperiod in determining cannabis yields, with particular focus on the potential for light-emitting diodes (LEDs) to improve growth and reduce energy requirements. We propose that the vast amount of genomics data currently available for cannabis can be used to better understand the effect of genotype on yield. Finally, we describe diversification that is likely to emerge in cannabis growing systems and examine the potential role of plant-growth promoting rhizobacteria (PGPR) for growth promotion, regulation of cannabinoid biosynthesis, and biocontrol.

## Introduction: Changing Attitudes on Cannabis and Current Knowledge Gaps

Currently cannabis laws are changing rapidly around the world, with legalization of medical use appearing in many jurisdictions, followed by legalization of recreational use. In Canada, this has led to significantly lower barriers to obtaining a license to conduct scientific research under the newly adopted Cannabis Act, in comparison with the Access to Cannabis for Medical Purposes Regulations (ACMPR) and its predecessor acts: Marihuana for Medical Purposes Regulations (MMPR) and Marihuana Medical Access Regulations (MMAR) (Canada, 2001, 2013, 2016, 2018). However, in the United Sates, while recreational cannabis has been legalized in nine states and medical cannabis has been legalized in 21 states (http://www.governing.com/gov-data/safety-justice/state-marijuana-laws-map-medical-recreational.html), cannabis remains illegal at the federal level, presenting a major barrier to research. To meet projected demand for medical and recreational cannabis products, the yield gap must be closed with the use of modern scientific tools.

Cannabis is one of the oldest cultivated crops and is used for food (seeds), fiber (stems), and drugs (flowers); it was domesticated in Central Asia over 6,000 BCE (Li, 1973; Mercuri et al., 2002; Clarke and Merlin, 2013, 2016). This genus produces over 200 secondary metabolites, including terpenes, phenolic acids and cannabinoids (Andre et al., 2016). In particular, medical and recreational cannabis are cultivated for, tetrahydrocannabinol (THC), and cannabidiol (CBD), which produce physiological and intoxicating effects in humans, which have been associated with both positive and negative health outcomes (Hill et al., 2012; Giacoppo et al., 2014; Volkow et al., 2014; Burstein, 2015; Van Amsterdam et al., 2015). Because cannabis naturally contains THC and CBD, this plant has been listed as a controlled substance for the last several decades in jurisdictions worldwide. Restrictions around cultivation of this plant has led to a void of scientific research.

**Figure 1:**
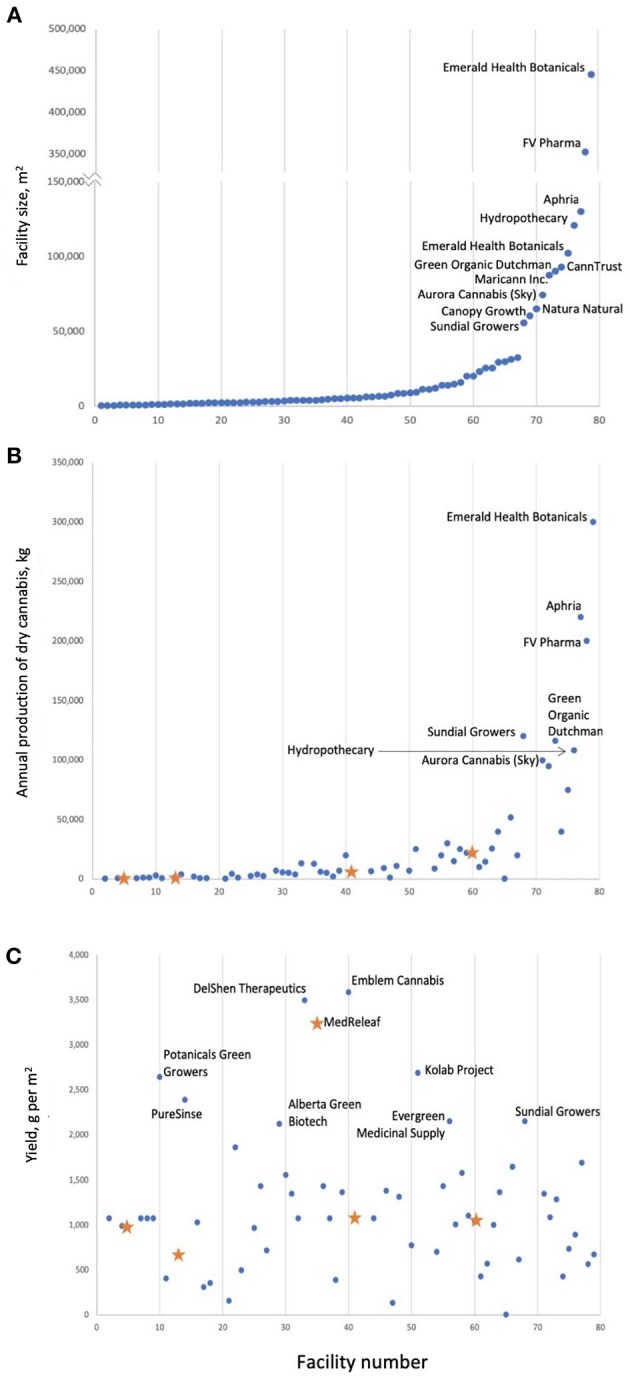
Cannabis production in Canada; facilities are numbered by facility size **(A)**. Annual production tends to increase with facility size **(B)** not yield per square meter **(C)**. It is important to note that it is unclear if facility size is always equal to the area of the cannabis production space. Blue dots are projected yields; orange stars are actual yields and correspond to AB Labs (Facility #5), United Greeneries (Facility #13), MedReleaf (Facility #35), Mettrum (Facility #41), WeedMD (Facility #60), and Canopy Growth (Facility #69). Values are current as of April 2018.

For cannabis, the yield gap constitutes the difference between the maximum possible flower yield compared to current yields obtained in commercial production. In addition, there is the important consideration of cannabinoid concentration and profile, which together determine the quality of the product. Legal cannabis-producing operations in Canada, show projected yields that range from 3.36 to 3590 g dry flower m^−2^ (Figure 1, Table S1) with MedReleaf achieving the highest yields per square meter. The first question that must be answered is: is this the physiological maximum of cannabis plants? The second question is which production conditions lead to obtaining these high yields? Another point requiring clarification is whether the most important yield is in fact the dry flowers (which contain the highest concentration of medicinal compounds) or the whole plant (for extraction of medicinal compounds, even from stems and leaves, which contain significantly lower concentrations).

To date, a limited number of studies have examined factors contributing to the cannabis yield gap. First, a body of literature has developed to provide a detailed knowledge base about existing cannabis strains, at the molecular level. Studies have begun to elucidate the genetic structure and diversity of cannabis (Sawler et al., 2015; Welling et al., 2016a), understand the inheritance of chemotype (De Meijer et al., 2003), and to catalog existing cannabis strains based on metabolomic fingerprinting methods and chemotaxonomy (Hazekamp et al., 2004, 2016; Hillig, 2004; Hillig and Mahlberg, 2004; Fischedick et al., 2010; Hazekamp and Fischedick, 2012). Some, but substantially less, research has investigated the impact of production methods on yield and cannabinoid profiles. This includes a study on the use of microbial inoculants (Winston et al., 2014), the role of light intensity and photoperiod (Chandra et al., 2011a, 2015), temperature (Chandra et al., 2011b), fertilization (Malceva et al., 2011; Caplan et al., 2017), physiological stresses (Lydon et al., 1987; Marti et al., 2014) and elicitors (Flores-Sanchez et al., 2009; Mansouri et al., 2009a,b, 2011, 2013, 2016; Mansouri and Asrar, 2012). These strategies have all played an important role in closing the yield gap in other crops and should be considered a good starting point for cannabis research.

In this meta-analysis, we examine the role of plant variety (genotype) and production conditions (plant density, light, fertilizer, temperature and duration of the flowering growth stage) on yield per plant, per square meter and per W of lighting, and THC and CBD yield per plant and per square meter. We describe currently available genomics and transcriptomics data for cannabis and how these can be used to produce a better understanding of the cannabis plant. We also examine the role of production conditions in predicting plant yields and examine the potential use of light emitting diodes and plant growth promoting rhizobacteria as novel production methods for obtaining high yields.

## Materials and Methods

### Data Collection

Data were collected as treatment means, based on variety, plant density (plants m^−2^), concentration of CO_2_ during cultivation, light intensity (W m^−2^ and photosynthetically active radiation, PAR, μmol m^−2^ s^−1^), light source (high pressure sodium, HPS, or fluorescent), photoperiod during vegetative growth and flowering stage (h), maximum temperature during growth (°C), and fertilizer rate (mg N L^−1^) from Vanhove et al. (2011, 2012), Potter and Duncombe (2012), Potter (2014), Caplan et al. (2017) and Conant et al. (2017) (Table S2). Based on availability, yield was recorded as either yield per plant, yield m^−2^ and/or yield W^−1^; percent THC and CBD in flowers at harvest were also recorded (Table S1). For data obtained from Potter and Duncombe (2012), yield m^−2^ was calculated by multiplying yield W^−1^ (g W^−1^) by light intensity (W m^−2^); yield plant^−1^ was calculated by multiplying yield m^−2^ by plant density (plants m^−2^). For data obtained from Vanhove et al. (2011, 2012), yield W^−1^ was calculated by dividing yield m^−2^ (g m^−2^) by light intensity (W m^−2^). For data obtained from Vanhove et al. (2011) and Potter and Duncombe (2012) THC yield (mg plant^−1^) was calculated by multiplying the proportion of THC in plant material (percent divided by 100) by the yield plant^−1^ (mg). For data obtained from Vanhove et al. (2011), THC yield m^−2^ (mg m^−2^) was calculated by multiplying the proportion of THC in plant material (percent divided by 100) by the yield m^−2^ (mg). For data obtained from Vanhove et al. (2011), CBD yield (mg plant^−1^) was calculated by multiplying the proportion of CBD in plant material (percent divided by 100) by the yield plant^−1^ (mg) and CBD yield m^−2^ (mg m^−2^) was calculated by multiplying the proportion of CBD in plant material (percent divided by 100) by the yield m^−2^ (mg). For yield W^−1^ data obtained from Potter and Duncombe (2012), data was extracted from figures using WebPlotDigitizer software (available at https://apps.automeris.io/wpd/).

### Modeling Approach

Data used for analysis can be found in Table S3. All analyses were conducted using SAS 9.4 (SAS Institute Inc. 2013). Variables with excessive missingness (CO_2_ concentration (ppm), light intensity (PAR μmol m^−2^ s^−1^), fertilizer rate or inoculation with Mammoth P^TM^) were not considered. T_max_, photoperiod during vegetative growth and duration of the vegetative or flowering periods were highly correlated to other variables (|*r*| > 0.75) and were therefore not included in the analysis. Prior to analysis, the remaining variables were standardized (mean = 0 and standard deviation = 1) using PROC STANDARD and categorical variables (light type, fertilizer type or variety) were recoded as binary variables (0 or 1).

PROC REG, with the SELECTION = STEPWISE option, was used to stepwise select variables. The list of unselected variables included the experimental continuous variables (plant density, light intensity, duration of the flowering period, and pot size) and their squared effects, categorical variables (light type, fertilizer type, and variety), and the cross-products between the continuous and categorical variables. Models were then constructed using PROC GLIMMIX with stepwise selected variables. A distribution to model the residuals was selected by comparing model fit statistics between gamma, inverse Gaussian, shifted-*t* distribution, exponential, normal, and lognormal distributions and the model with the lowest Bayesian information criterion was selected. A random component was added to account for the source of the data. Components of the models that were not statistically significant (*F*-test *p* > 0.05) were removed sequentially until all variables remaining in the model were statistically significant. In some models, numerical class variables were classified as categorical variables to produce estimates for least squares-means.

## Results

Models were constructed to describe yield plant^−1^, yield m^−2^, yield W^−1^, THC and CBD yield plant^−1^ and m^−2^. Given the high correlations, the effects of density cannot be separated from the effects of maximum temperature during cultivation and the photoperiod used during the vegetative growth period. Therefore, the effect of maximum temperature is interpreted as having the same effects as plant density, whereas the vegetative photoperiod had the inverse effect as density. Likewise, the effects of maximum temperature and duration of the vegetative growth period have effects that are the inverse of flowering duration effect. Because yield m^−2^ and W^−1^, THC m^−2^ and CBD m^−2^ are most relevant for industry, those results are highlighted here. Formulae to predict yield, THC and CBD plant^−1^ are found in the Supplementary File.

**Figure 2:**
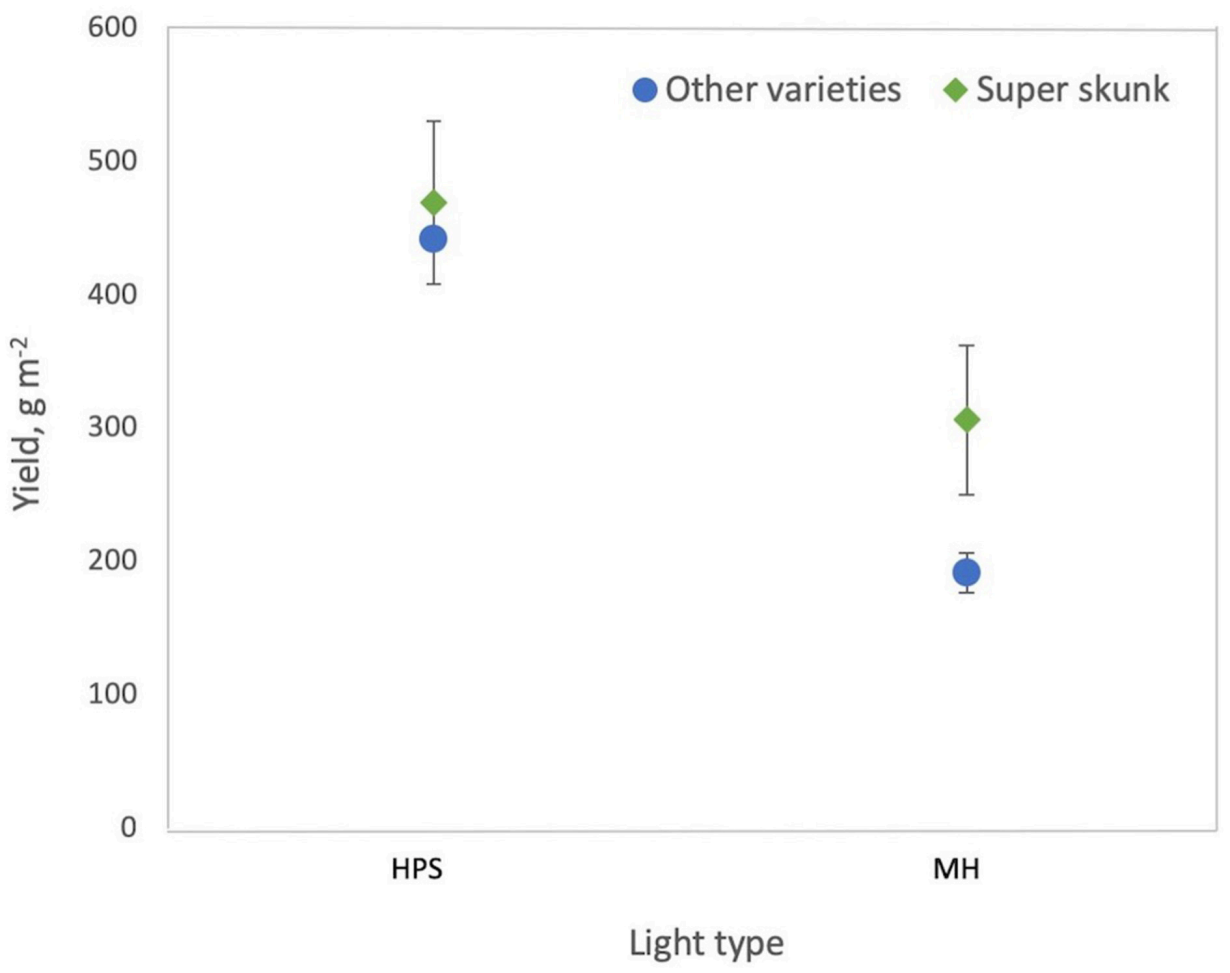
Effect of light type on cannabis yield per square meter. High pressure sodium (HPS) lamps produce higher yields than metal halide (MH) lamps and Super Skunk plants produce higher yields than other varieties when grown under MH lamps.

**Figure 3:**
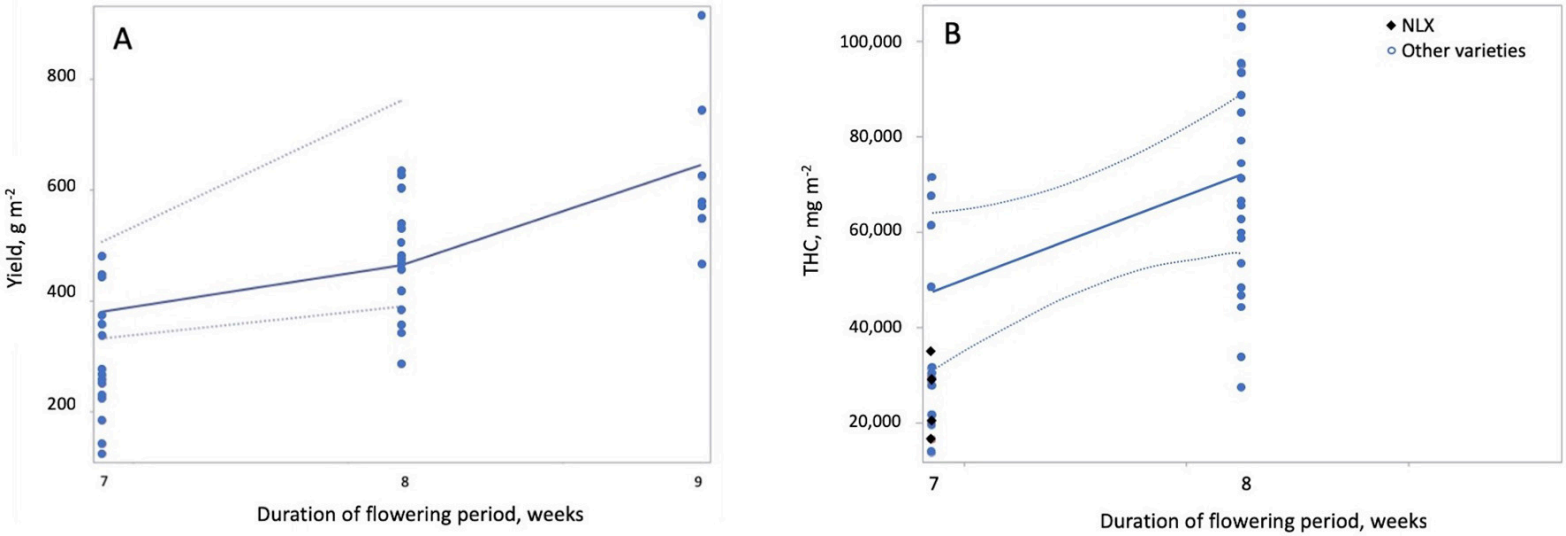
Effect of the duration of the flowering growth period on yield and THC per square meter. Both yield per square meter **(A)** and THC per square meter **(B)** increased with increasing duration of the flowering period. Duration of the flowering period had a strong (|r| > 0.7) negative correlation to maximum temperature and duration of the vegetative growth period; therefore, these predictors have the opposite effects on yield and THC per square meter as duration of the flowering period.

Based on the studied data, yield m^−2^ can be predicted using the formula:

$$\begin{array}{l} \frac{1}{{\left(Yield\;m^{-2}\right)}^{2}}=5.136\times 10^{-6}+\left(-1.66\times 10^{-6}\times F_{dur}\right)\\ \;+\;\left(\begin{array}{lll} L_{type} & V_{SS} & b_{2}\\ 1 & 1 & 5.545\times 10^{-6}\\ 1 & 0 & 0.00022\\ 0 & 1 & -5.78\times 10^{-7}\\ 0 & 0 & 0 \end{array}\right) \end{array}$$

where *F*_*dur*_ is duration of the flowering period on the statistically standardized scale, *L*_*type*_ is light type (where 0 = HPS and 1 = MH) and *V*_*SS*_ = 1 indicates Super Skunk. For varieties other than Super Skunk, plants grown under HPS lamps had higher yields m^−2^ than plants grown under MH lamps (*p* < 0.0001) and for other varieties grown under MH lamps, yields from Super Skunk plants were higher than for all other varieties (*p* = 0.0058) (Figure 2). Yield m^−2^ increased with increasing duration of the flowering period (*p* = 0.0005) (Figure 3A).

**Figure 4:**
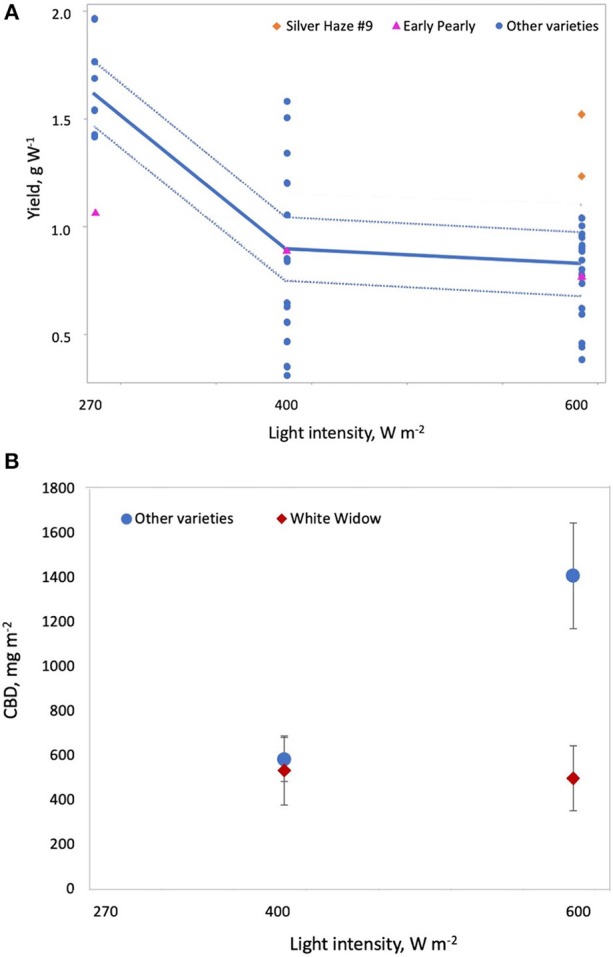
Effect of light intensity on cannabis yield per W and CBD per square meter. **(A)** Increasing light intensity reduces yield per W and this effect is stronger for most varieties other than Early Pearly and Silver Haze #9, which maintained higher yields at 600 Wm^−2^. **(B)** Varieties other than White Widow produced significantly more CBD at 600 Wm^−2^ compared to 400 Wm^−2^.

**Figure 5:**
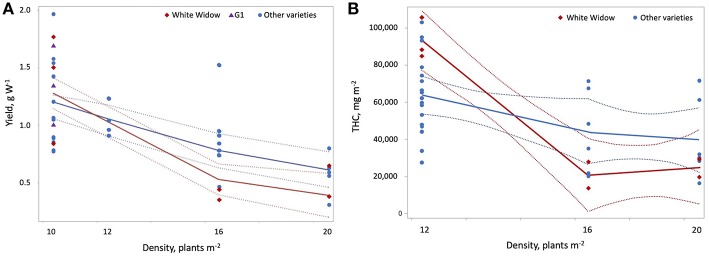
Effect of plant density on yield per W and THC per square meter. Yield per W **(A)** and THC per square meter **(B)** declined with increasing plant density. These effects were stronger for White Widow than for other varieties of cannabis. G1 had higher yields per W compared to other varieties at a plant density of 10. Plant density had a strong (|r| > 0.7) positive correlation with maximum temperature during cultivation and a strong negative correlation with the duration of the vegetative photoperiod.

**Figure 6:**
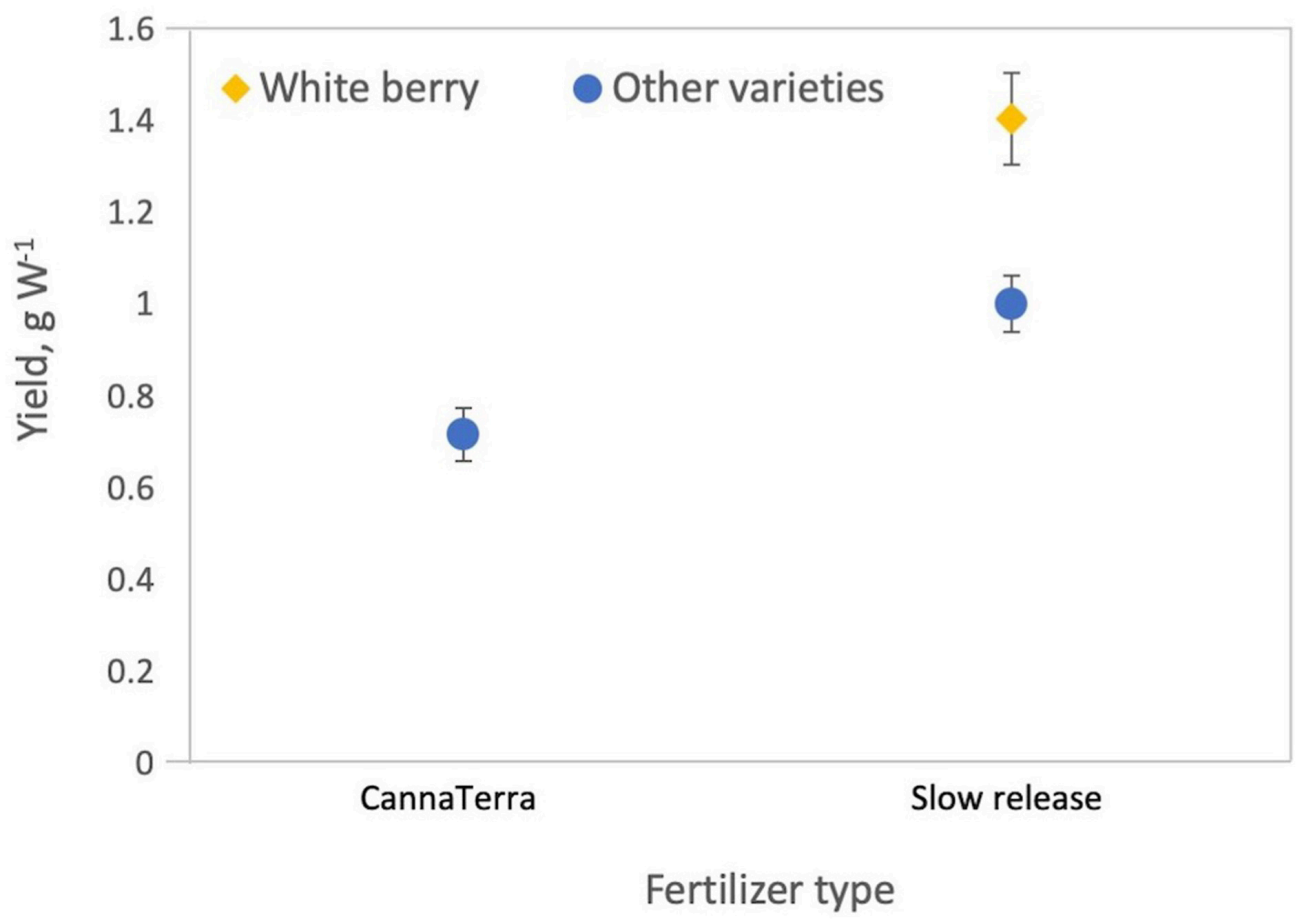
Slow release fertilizer produced higher yields per W compared to the CannaTerra fertilizer regime. When slow release fertilizer was applied, White Berry produced higher yields per W compared to other varieties.

Yield W^−1^ can be predicted using the formula:

$$\begin{array}{l} Yield\;W^{-1}=1.0032+\left(D\times \left(\begin{array}{cc} V_{G1} & b_{1}\\ 1 & -0.2258\\ 0 & 0.04358 \end{array}\right)\right)\\ \;+\left(D\times \left(\begin{array}{cc} V_{WW} & b_{2}\\ 1 & -0.1799\\ 0 & 0 \end{array}\right)\right)+\left(L_{int}\times \left(\begin{array}{cc} V_{SH9} & b_{3}\\ 1 & 0.2119\\ 0 & 0 \end{array}\right)\right)\\ \;+\left(L_{int}\times \left(\begin{array}{cc} V_{EP} & b_{4}\\ 1 & 0.2192\\ 0 & 0 \end{array}\right)\right)+\left(L_{int}\times \left(\begin{array}{cc} F_{type} & b_{5}\\ 1 & 0.3377\\ 0 & 0 \end{array}\right)\right)\\ \;+\left(F_{D}\times \left(\begin{array}{cc} V_{NLX} & b_{6}\\ 1 & 0.2747\\ 0 & 0.09662 \end{array}\right)\right)+\left(\begin{array}{ccc} F_{type} & V_{WB} & b_{7}\\ 0 & 0 & -0.2848\\ 1 & 1 & 0.4036\\ 1 & 0 & 0 \end{array}\right) \end{array}$$

where *D* is plant density on the statistically standardized scale, *V*_*G*1_ = 1 indicates variety G1, *V*_*WW*_ = 1 indicates White Widow, *L*_*int*_ is light intensity on the statistically standardized scale, *V*_*SH*9_ = 1 indicates Silver Haze #9, *V*_*EP*_ = 1 indicates Early Pearly, *F*_*type*_ is fertilizer type (where 0 = CannaTerra and 1 = slow release fertilizer), *F*_*D*_ is duration of the flowering period on the statistically standardized scale, *V*_*NLX*_ = 1 indicates Northern Lights #5 × Haze and *V*_*WB*_ = 1 indicates White Berry. Increasing light intensity reduced yield W^−1^ but Silver Haze #9 produced higher yields W^−1^ compared to other varieties at 600 W m^−2^ and Early Pearly was less sensitive to this decrease compared to other varieties (*p* = 0.0006 and *p* = 0.0099, respectively) (Figure 4A). While increasing plant density reduced yield W^−1^, the effect was slightly different for G1 and White Widow compared to other varieties (*p* = 0.0133 and *p* = 0.0042, respectively) (Figure 5A). Yield W^−1^ was higher for plants grown using slow release fertilizer compared to the CannaTerra nutrient regime (*p* < 0.05) and when slow release fertilizer was applied, White Berry had higher yield W^−1^ than other varieties (*p* < 0.05) (Figure 6). For plants fertilized with CannaTerra, increased light intensity increased yield W^−1^ (*p* < 0.0001). Yield W^−1^ increased with flowering duration and this effect was stronger for the variety Northern Lights #5 × Haze than other varieties (*p* = 0.0013).

**Figure 7:**
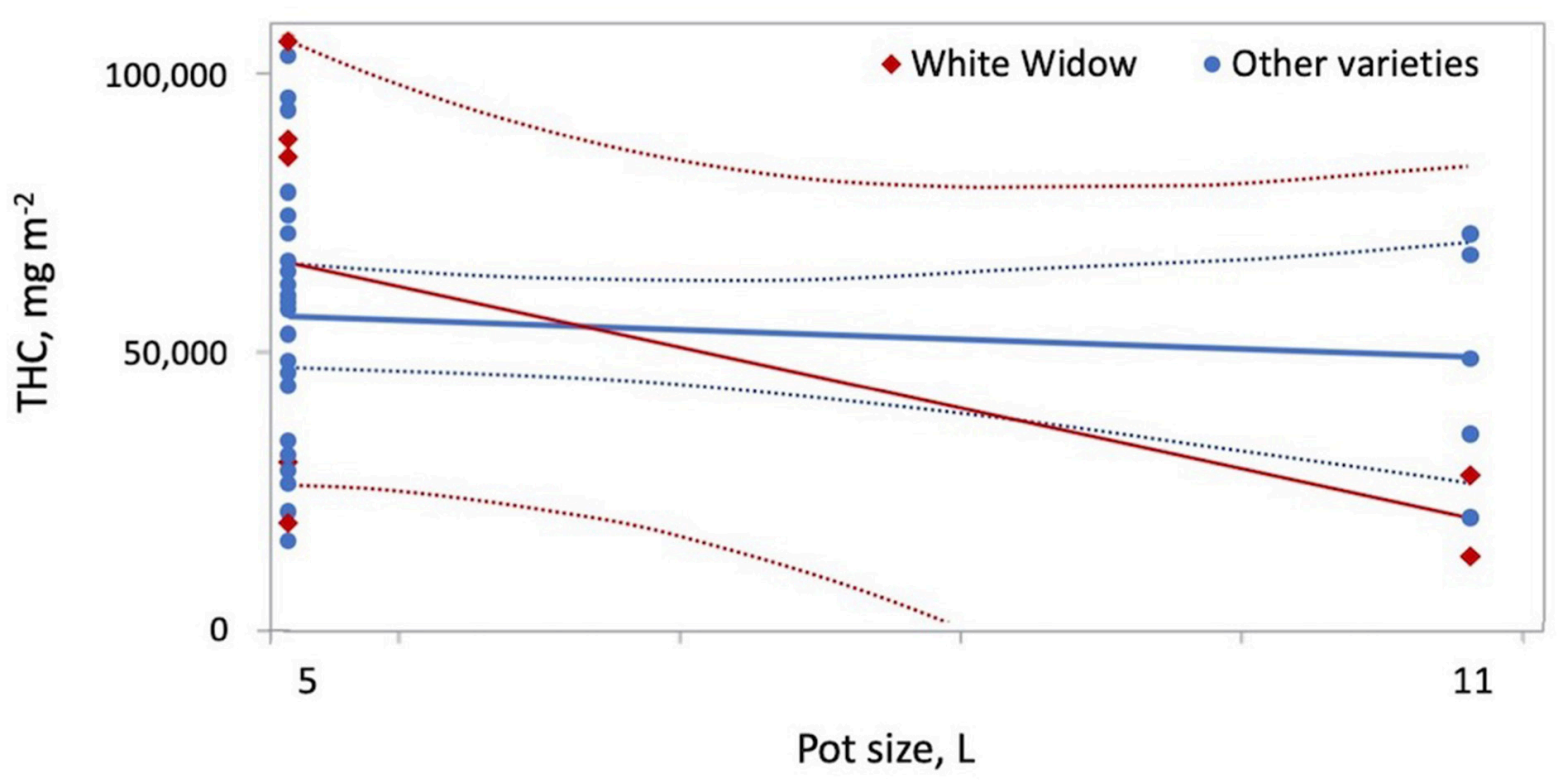
Increasing pot size from 5 to 11 L reduced THC per square meter more for White Widow compared to other varieties.

THC per m^−2^ can be described according to:

$$\begin{array}{l} \ln\left(THC\;m^{-2}\right)=11.1634+\left(0.1397\times L_{int}\right)+\left(D\times \left(\begin{array}{cc} V_{Wa} & b_{2}\\ 1 & -0.1108\\ 0 & 0.2274 \end{array}\right)\right)\\ \;+\left(D\times \left(\begin{array}{cc} V_{WW} & b_{3}\\ 1 & -0.3824\\ 0 & 0 \end{array}\right)\right)+\left(L_{int}\times \left(\begin{array}{cc} V_{BB} & b_{4}\\ 1 & 0.4040\\ 0 & 0 \end{array}\right)\right)\\ \;+\left(F_{D}\times \left(\begin{array}{cc} V_{NLX} & b_{5}\\ 1 & 1.2069\\ 0 & 0.7397 \end{array}\right)\right)+\left(P_{s}\times \left(\begin{array}{cc} V_{WW} & b_{6}\\ 1 & -0.1676\\ 0 & 0.1735 \end{array}\right)\right)\\ \;+\left(\begin{array}{ccc} V_{EP} & F_{type} & b_{7}\\ 1 & 1 & -0.5042\\ 0 & 0 & 0\\ 0 & 1 & 0 \end{array}\right)+\left(\begin{array}{ccc} F_{type} & V_{SS} & b_{8}\\ 0 & 1 & 0.1692\\ 0 & 0 & 0\\ 1 & 1 & 0.4660\\ 1 & 0 & 0 \end{array}\right) \end{array}$$

where *L*_*int*_ is light intensity on the statistically standardized scale, *D* is the plant density on the statistically standardized scale, *V*_*Wa*_ = 1 indicates Wappa, *V*_*WW*_ = 1indicates White Widow, *V*_*BB*_ = 1 indicates Big Bud, *F*_*D*_ is the duration of the flowering period on the statistically standardized scale, *V*_*NLX*_ = 1indicates Northern Lights #5 × Haze, *P*_*S*_ is the pot size on the statistically standardized scale, *F*_*type*_ is fertilizer type (where 0 = CannaTerra and 1 = slow release fertilizer) and *V*_*EP*_ = 1 indicates Early Pearly. THC m^−2^ was lower at a light intensity of 400 W m^−2^ compared to 270 or 600 W m^−2^ (*p* = 0.0001) and this effect was stronger for Big Bud than for other varieties (*p* = 0.0116). Increasing the duration of the flowering period led to increased THC m^−2^ for varieties other than Northern Lights #5 × Haze (*p* = 0.0006). Increased plant density reduced THC m^−2^ for all varieties; this effect was stronger for White Widow than the other varieties (*p* = 0.0002) (Figure 5B). Increasing the pot size from 5 to 11 L reduced THC m^−2^ for White Widow but had a much smaller effect on other varieties (*p* = 0.0035) (Figure 7). Early Pearly produced lower THC m^−2^ compared other varieties when slow release fertilizer was applied (*p* = 0.0004) whereas for Super Skunk produced more THC m^−2^ compared to other varieties when either fertilizer was applied (*p* = 0.0017).

CBD m^−2^ can be described according to:

$$\begin{array}{l} \ln\left(CBD\;m^{-2}\right)=7.2498+\left(\begin{array}{ccc} L_{int} & V_{WW} & b_{1}\\ 400 & 1 & -0.9719\\ \begin{array}{c} 400\\ 600\\ 600 \end{array} & \begin{array}{c} 0\\ 1\\ 0 \end{array} & \begin{array}{c} -0.8820\\ -1.0370\\ 0 \end{array} \end{array}\right) \end{array}$$

where *L*_*int*_ is light intensity (W m^−2^) and *V*_*WW*_ = 1 indicates White Widow. White Widow responded differently to light intensity than other varieties (*p* = 0.0077); White Widow had lower CBD m^−2^ compared to other varieties at a light intensity of 600 W m^−2^, however this effect was not statistically significant (*p* > 0.05) (Figure 4B).

## Discussion

### Effect of Production Conditions on Yield and Cannabinoid Content

As highlighted in the data presented, yields obtained for cannabis are highly variable depending on variety, production conditions and production methods. Furthermore, these data highlight the discrepancy of yields obtained in industry compared to experimental settings. This stresses the importance of replicating industrial growing conditions in a research setting to allow for translation to the commercial grower setting. This applies equally to studies designed to enhance production based on traditional methods such as fertilization, lighting regimes, plant density and also to novel methods to be tested, including the use of plant-growth promoting rhizobacteria (PGPR) or LED-based lighting systems. This section describes what is currently known about cannabis cultivation in the scientific literature, with some references to industry norms, and also underlines areas of significant opportunity for scientific development relevant to the cannabis industry.

### Plant Density, Pot Size and Fertilizer Regime Affect Yield per W and THC Yield

The results of the meta-analysis highlight the impact of production conditions on cannabis yield per plant, per square meter and per W of lighting. While increasing plant density reduced yield per W and THC per square meter, plant density was not an effective predictor of yield per square meter (Figure 5). The experimental designs used cannot quantify the relative contribution of increasing maximum temperature and/or shortening of the vegetative photoperiod compared to increasing plant density; these factors should be studied in more detail in future experiments. Furthermore, Chandra et al. (2008) recorded a maximum rate of photosynthesis for *C. sativa* grown at 30°C, compared to plants grown at 20–40°C, which explains how yield per square meter are maintained even at higher temperatures. Furthermore, the slightly stressful conditions of increased plant density and maximum temperature may contribute to increased THC accumulation. Previously, accumulated THC increased in response to the application of abscisic acid, a plant stress hormone (Table 1) (Mansouri et al., 2009a, Mansouri et al., 2012). Increasing pot size reduced THC per square meter, especially for the variety White Widow (Figure 7).

**Table 1:** Elicitors that have been tested on cannabis and their effects on secondary metabolite concentrations, in particular THC and CBD.

**Elicitor**	**Elicitor concentration**	**Main result**	**Form of *C. sativa***	**References**
Yeast extract	10 mg mL^−1^	Shifts in metabolites were observed but cannabinoid biosynthesis appeared to be absent	Hairy root cell culture	Flores-Sanchez et al., 2009
*Pythium aphanidermatum*	4 and 8 g mL^−1^			
*Botrytis cinerea*	4 and 8 g mL^−1^			
Salicylic acid	0.3, 0.5, 1 mM			
Methyl jasmonate	0.3 mM			
Jasmonic acid	100 μM			
Cannabis pectin extract	84 μg mL^−1^			
Cannabis pectin hydrolyzed	2 mL aliquot			
Pectin	0.1 mg mL^−1^			
Sodium alginate	150 μg mL^−1^			
AgNO_3_	50 and 100 μM			
CoCl_2_-6H_2_O	50 and 100 μM			
NiSO_4_-6H_2_O	50 and 100 μM			
UV 302 nm	30 s			
UV 366 nm	30 min			
Absisic acid	1, 10 mg L^−1^	Increased THC	Whole plants	Mansouri et al., 2009b
	1, 10 μM	Increased cannabichrome, cannabinol	Whole plants	Mansouri and Asrar, 2012
Cycocel	500, 1000, 1500 mg L^−1^	Increased/decreased THC, CBD depending on tissue, treatment concentration, plant sex	Whole plants	Mansouri and Rohani, 2014
Ethephon	1, 5, 10, 100 μM	Increased cannabinoids in male and female plants	Whole plants	Mansouri et al., 2013
	1, 5, 10, 100 μM	Increased THC, decreased CBD	Whole plants	Mansouri et al., 2016
Gibberellic acid	5, 10, 30, 70, 100 μM	Increased THC, CBD	Whole plants	Mansouri et al., 2011
	50, 100 μM	Decreased THC	Whole plants	Mansouri et al., 2009a
Mevinolin	0.1, 1, 10 μM	Decreased THC	Whole plants	Mansouri and Salari, 2014

Interestingly, fertilizer type (CannaTerra compared to slow release fertilizer) affected yield per W (Figure 6) and THC per square meter but did not affect yield per plant or per square meter. This result are likely due to differences in nutrient concentration, the balance of plant nutrients, timing of application highlighting the important need to develop adequate nutrient regimes for cannabis. Caplan et al. (2017) provided the first publication on this topic and demonstrated that when a liquid organic fertilizer (4.0N - 1.3P - 1.7K) was applied at a rate of 389 mg N L^−1^ and 418 mg N L^−1^ during the vegetative growth stage, yield and THC concentration in dry flower biomass were optimized, respectively, for container-grown “OG Kush × Grizzly” plants on two coir-based substrates. Future studies should examine the effects of individual plant nutrients and their interactions on crop and cannabinoid yields, and studies should be expanded to include a wider range of cannabis growth stages, varieties and growing substrates.

### Light Intensity, Quality, and Duration of the Flowering Period Affect Flower and Cannabinoid Yield

Yield per square meter was higher when HPS lamps were used than when MH lamps were used (Figure 2). This is likely due to the lower luminous efficiency (i.e., lower light output per W) for MH lamps than HPS lamps (Eichhorn Bilodeau et al., 2019). This results in lower photosynthetic photon flux density (PPFD) for MH than HPS lamps, even if the W m^−2^ of the lamp is equivalent. THC and CBD per square meter increased with light intensity while yield per W decreased with increasing light intensity (Figure 4). The increased accumulation of THC and CBD at 600 W m^−2^ suggest that these compounds are produced to limit the effects of light stress at higher light intensities as a result of a stress response (Mansouri et al., 2009b; Mansouri et al., 2012). Our results also clearly indicate that increasing the duration of the flowering period (or reducing the duration of the vegetative period) increases yield per square meter and THC per square meter (Figure 3), a result which Vanhove et al. (2012) attributed to increased photosynthetic assimilation directed to bud growth instead of stem and leaf growth.

Light quality, intensity, source and photoperiod play a critical role in yield and quality of cannabis. Often, yield is reported as g W^−1^, as a measure of energy efficiency of the growing system. Literature values report yields of 0.3122–1.972 g W^−1^, and are influenced by strain, light intensity and plant density (Toonen et al., 2006; Vanhove et al., 2011, 2012; Potter and Duncombe, 2012). Furthermore, plants, including cannabis, are sensitive to the spectral composition of their source of light, which elicits specific effects on photosynthesis, photomorphogenesis, phototropism, and photonasty (Tamulaitis et al., 2005; Hogewoning et al., 2010). Use of electrical lighting systems with different spectral outputs is common in plant research and greenhouse horticulture. Most commonly, high pressure sodium (HPS) gas discharge lamps and fluorescent tubes are used (Hogewoning et al., 2010). Although the spectral emissions of these lights span the entire spectrum of sunlight, they feature distinct wavelength patterns (Hogewoning et al., 2010). HPS lights generally emit light most strongly in the yellow-red end of the spectrum, which is absorbed by chlorophyll and used in photosynthesis. Improvements in the blue component of HPS lights can improve light suitability for plant growth, however modifications are required to optimize the red spectrum of their emissions to enhance plant growth (Tamulaitis et al., 2005). These changes would reduce the energy lost as infrared radiation or heat. In contrast, fluorescent tubes have peaks throughout the spectrum but lack emissions in the far-red region of the spectrum (Tamulaitis et al., 2005). High power light emitting diodes (LEDs) are an emerging versatile electrical light source offering many advantages over conventional electrical light sources, including high energy efficiency, long life, and especially, the possibility to test the effects of many spectral combinations of wavelengths on plant growth and development. This could eventually lead to determination of the ideal light emission spectrum, allowing for lighting system designs tailored to enhance plant growth while minimizing associated energy costs (Tamulaitis et al., 2005). In the meantime, studies have begun to exploit the spectral elasticity of LEDs to examine the effects of different wavelength-light combinations on plant growth. The possibility of achieving higher irradiance at isolated wavelengths of light than with monochromatic light previously obtained through filters, could allow more accurate assessments of plant physiological responses (Lefsrud et al., 2008).

The optimal spectrum of light to achieve optimal yields of cannabis and cannabinoids remains to be fully elucidated. Environmental factors, such as temperature and irradiance levels, can have strong effects on plant growth and the accumulation of pigments critical for photosynthesis (Lefsrud et al., 2005, 2006). Chandra et al. (2008) discussed photosynthetic and water-use efficiency responses of cannabis to light, CO_2_ and temperature levels. The study demonstrated that maximum rate of photosynthesis occurs at 30°C, 750 μmol CO_2_ mol^−1^, and under 1,500 μmol m^−2^ s^−1^. The study concluded that high intensity lighting, in drier and CO_2_ enriched environments promotes higher photosynthetic activity, water use efficiency, and nearly constant internal to ambient CO_2_ concentration in cannabis.

Another challenge associated with lighting systems is that light intensity decreases with depth within the plant canopy as leaves absorb the light (Massa et al., 2005). In HPS and overhead LED lighting systems, the top of the canopy is often light saturated, yet the canopy as a whole is light limited. Providing additional light to the lower canopy increases the proportion of light used for photosynthesis without exceeding the point of photosynthetic light saturation (Massa et al., 2005). Unlike HPS lamps, LEDs emit little heat and can be placed close to the crop without burning leaves, meaning they are a practical interlighting system in commercial settings. For example, LEDs located within a cowpea (*Vigna unguicultata* L. Walp.) canopy improved biomass production by 33 %, compared to plants grown under overhead lights; intercanopy lights were also associated with an increased energy conversion rate (Massa et al., 2005). Hawley (2018) demonstrated that supplemental sub-canopy lighting (SCL) can increase cannabis bud yield and modify cannabinoid and terpene profiles. The increase in bud yield is assumed to be related to increased photosynthetic photon flux densities (PPFD) compared to production with overhead lighting alone. Red and blue SCL yielded a more consistent metabolite profile throughout the canopy, whereas red, green and blue SCL had the greatest impact on metabolite upregulation. A light spectrum with comparatively more green light drove plants to produce more carotenoids to manage green wavelengths, and consequently up-regulated other related terpenes in the process.

The effects of LEDs on plant growth and photomorphogenesis has been studied in plant species other than cannabis, with emphasis on the control of flowering and/or the duration of the blooming period. Physiological studies have shown that light quality, quantity and duration regulate flowering (Bula et al., 1991; Tennessen et al., 1994). According to Guo et al. (1998) and Thomas and Vince-Prue (1996), red light can inhibit flowering *via* red-light receptors such as phytochromes, which absorb light effectively at wavelengths above 600 nm (Kelly and Lagarias, 1985). In contrast, blue light can inhibit flowering *via* blue-light receptors such as cryptochromes, which absorb light well at wavelengths below 500 nm (Lin et al., 1995; Banerjee et al., 2007; Eichhorn Bilodeau et al., 2019).

A study on cannabis demonstrates that flowering time is determined by photoperiod: flowering is induced when day length is shorter than 12 h (Potter, 2014). While light quality influences on cannabis flowering have not yet been studied, light quality has been shown to influence flowering and duration of the blooming period in marigold (*Tagetes erecta* L. cv. Orange Boy) and salvia plants (*Salvia splendens* F. Sello ex Ruem & Schult. cv. Red Vista) (Heo et al., 2002). The number of visible flower buds in marigold was approximately five times higher in the presence of fluorescent light (with or without red LED) than under monochromic blue or red light. Monochromic blue or red light were found to suppress bud formation in salvia while fluorescent light plus far-red light was also found to inhibit flower bud formation in marigold. Day-extension using red or blue LEDs inhibited flower and bud appearances. Night-break treatment with red LEDs also delayed flower bud appearance in okra (*Abelmoschus esculentus* L. Moench) and a cultivar of native rosella (*Abelmoschus moschatus* ssp. tuberosus Span Borss). Night break with green light delayed flowering more strongly than blue light, but slightly less than red light (Hamamoto and Yamazaki, 2009). In long-day plants, experiments suggest that flowering is promoted most when red light is delivered during the early part of the photoperiod and far red light toward the end of the photoperiod (Lane et al., 1965; Evans, 1976; Kadman-Zahavi and Ephrat, 1976; Thomas and Vince-Prue, 1996). However, cannabis is a short-day plant, so it remains unclear whether these results are relevant for cannabis production.

### Effect of Variety on Crop Yield and Cannabinoid Content

The results of the meta-analysis show that yield per square meter and per W and accumulation of THC and CBD vary based on plant variety. Sawler et al. (2015) showed that variety name does not always correspond to genotype, as so it is critical that future reports, document the genotype used to allow for comparison of results from different studies. It is also worth highlighting that while Silver Haze #9 stands out as a top-yielding variety, it was pruned differently than other varieties included in the same study. Therefore, the high yields of this variety may be related to pruning rather than to its genotype and both possibilities should be investigated in future research. Our results confirm the findings of Vanhove et al. (2012), who showed that varieties respond differently to changes in production conditions, as evidenced by multiple significant variety-by-production condition interaction effects.

### *Cannabis* Genetic and Chemical Diversity

Cannabis plants are be classified as *indica, sativa*, and *ruderalis*. Lack of scientific consensus means these terms refer to cannabinoid content, morphology, allele frequencies or provenance (Hillig, 2005; Dufresnes et al., 2017). Historically, hemp-type (high in cannabidiolic acid, CBDA) and medical/recreational-type (often called marijuana, high in tetrahydrocannabinolic acid, THCA) strains have been categorized by their chemotype. For example, hemp is legally defined by EU and Canadian regulations as containing <0.3% THC (Canada, 1998). Species level classification of *Cannabis* plants is complicated by the lack of reproductive barriers between individuals conventionally described as subspecies, phenotypic plasticity, strong artificial selection for fiber-type and drug-type plants, as well as mixing of wild and cultivated populations since antiquity (Sawler et al., 2015; Clarke and Merlin, 2016; Grassa et al., 2018). More recently, genomic and transcriptomic distinctions between hemp and medical/recreational cannabis have been made (Piluzza et al., 2013; Sawler et al., 2015). Sawler et al. (2015) identified ~14,000 single-nucleotide polymorphisms that distinguished hemp-type and medical/recreational-type plants. Welling et al. (2016a) used genomic markers to predict the cannabinoid profile of 22 *Cannabis* accessions with over 98% accuracy, thereby confirming the genetic underpinning of chemotype.

The *Cannabis* genome is diploid (2n = 20) with nine autosomal chromosome pairs and one pair of XY sex chromosomes (Sakamoto et al., 1998; Divashuk et al., 2014). The nuclear genome was characterized and determined to be ~1,636 Mb for female plants (XX) and 1,683 Mb for male plants (XY) (Sakamoto et al., 1998). In 2011, the first draft haploid genome sequences were published (Van Bakel et al., 2011). These included a female clone of the drug-type cultivar Purple Kush, and a female plant of the fiber-type cultivar Finola (Van Bakel et al., 2011). Theses genomes were assembled from Illumina paired-end (6 libraries with median insert sizes ranging from 220 to 600 bp), Illumina mate-pair (2 libraries with median insert sizes of 1.8 and 4.6 kb), and 454 mate-pair (11 libraries with median insert sizes ranging from 8 to 80 kb) libraries (Van Bakel et al., 2011). The assembled Purple Kush genome was 786.6 Mb including 252 Mb of gaps. The presence of gaps in the genome was attributed to high repeat content and to high sequence variation in the cannabis genomes. More recently, an ultra-high-density genetic map was generated for Cannabis using a combination of long and short read sequencing technologies across parental, F1, and 96 recombinant F2 individuals (Grassa et al., 2018). Long-read technologies, including those from PacBio, have been used to sequence through repetitive regions, in order to close sequencing gaps in a number of plant species. Several long-read cannabis genome sequences have been contributed to the NCBI Genome repository. None of these sequences are associated with peer-review publications, nor are they presented as assembled or annotated genomes. Additionally, more than 1,500 short-read genome sequencing samples have been deposited in NCBI, including whole genome sequences, genotype by sequence, and short read assemblies. Many of these accessions are not associated with publications, and lack metadata to permit their full use by the research community. In spite of the lack of metadata, these genome accessions can be used to examine variation in the genomes of a range of cannabis cultivars.

The first published cannabis transcriptomes were synthesized from the roots, stems, vegetative shoots, pre-flowers and flowers of Purple Kush; more than 18.8 Gb of poly-A+ RNA reads corresponding to 30,000 genes were identified (Van Bakel et al., 2011). Since then, a leaf tissue salinity response transcriptome has also been published (Liu et al., 2016). A slightly larger number of transcriptome studies exist for hemp-type cannabis plants (Behr et al., 2016; Booth et al., 2017; Guerriero et al., 2017). However, the functional characterization of the cannabis genome is still in its infancy.

#### Crop Improvement Using Genomics and Transcriptomics

The diverse uses of cannabis plants are reflected in the significant variation in their stalk height, seed size, fiber length, phytochemical concentrations, and sensitivity to day length (Clarke and Merlin, 2016). Many of these traits, including those typically attributed to *indica, sativa* and *ruderalis*-type plants (Gould, 2015), may be targeted for improvement using conventional or modern breeding technologies. Detailed knowledge of the variation that exists across the *Cannabis* genus is fundamentally important to any project aiming to improve cultivars. Several projects have characterized the genetic structure of small populations of cannabis (Gao et al., 2014; Sawler et al., 2015; Soorni et al., 2017), but this has not yet been done on a larger scale. This synthesis of the knowledge has not yet transpired as the illicit nature of the drug-type plant has delayed the establishment of a well-conserved and well-annotated germplasm with consistent nomenclature (Clarke and Merlin, 2016). There is a movement in the cannabis research community to preserve and analyze germplasm across the genus to facilitate research and breeding programs (Clarke and Merlin, 2016; Welling et al., 2016b; Small, 2018).

Starting in the 1990s, molecular markers for cannabis varieties were developed for forensic analysis of plant origin. Hemp breeders have since integrated molecular markers (namely sex-linked and chemotypic markers) to enhance marker-assisted selection (MAS) strategies for crop improvement (Mandolino and Carboni, 2004; Faux et al., 2016) and cannabis researchers have used QTL analysis to identify loci associated with THCA production (Weiblen et al., 2015). A number of marker sets have been generated for a variety of genetic loci including microsatellites (Dufresnes et al., 2017) and SNPs associated with traits of interest (Sawler et al., 2015; Lynch et al., 2016; Soorni et al., 2017). Following the advent of next-generation sequencing, QTL mapping and genome-wide association studies have become more feasible, which will accelerate the discovery of important markers. Due to the high phenotypic plasticity of cannabis, associations between markers and phenotypes must be carefully characterized (Salentijn et al., 2015). The advent of genome editing technologies also hold great promise for cannabis improvement as *Agrobacterium* mediated transformation protocols have been published (Feeney and Punja, 2003).

Efficient genome editing capabilities facilitated by the biotechnologies of CRISPR-Cas9 and related technologies hold great promise for targeted improvement of Cannabis cultivars. For these technologies to be implemented three companion methodologies must be established: (1) micropropagation; (2) efficient transformation; (3) plant regeneration. Micropropagation technologies are foundational to the Cannabis industry, where they are used primarily with the aim of propagating and expanding high value cannabis plants. For the purposes of biotechnology applications, it is necessary to develop and maintain cultures pluripoten stem cells as callus or cell suspension culture. Since the 1970s, a number of such protocols have been established for Cannabis (reviewed in Lata et al., 2017; Wróbel et al., 2018). Transformation of Cannabis cells using *Agrobacterium tumafasciens* and *A. rhizogenes* have been demonstrated starting in 2003 with the transformation of callus (Feeney and Punja, 2003) and more recently using callus derived from a variety of tissue types and cultivars (Slusarkiewicz-Jarzina et al., 2005; Wahby et al., 2013). Protocols for the transformation of Cannabis roots have also been established (Wahby et al., 2013). The primary and persistent challenge has been to regenerate plants from the transformed callus and explant tissue (Feeney and Punja, 2003); plant recovery rates range from <2% to more than 50% (Chaohua et al., 2016) depending on the protocol, starting tissue, and genotype used. To date, we are not aware of any published accounts of CRISPR mediated genome editing in Cannabis; this will undoubtedly not be the case for long.

### Limitations of Available Cannabis Data

This meta-analysis was able to identify some key factors that contribute to cannabis yields. However, only three studies were included in the meta-analysis due to the fact that other published studies did not report sufficient information about growing conditions for inclusion in the models (Table S2). Furthermore, it remains difficult to determine the relationship between flower and cannabinoid yields due to the lack of consistent reporting of cannabinoid concentration or yield. Our results also show that light type, as a proxy for PPFD, has a significant impact on flower and CBD yields, which suggests that reporting of light intensity as W m^−2^ is insufficient on its own. Finally, the results of this meta-analysis show that yield per square meter obtained in scientific studies (Table S1) remains much lower than yield per square meter obtained in industry (Figure 1) suggesting that discrepancies remain between industry production practices and growing conditions used in scientific studies. This highlights the value of knowledge exchange between academia and industry.

### Future Considerations in Cannabis Research

#### Diversification of Cultivation Systems for Cannabis

Currently, cultivation of medical cannabis is usually conducted in controlled environment growing rooms since they offer a higher degree of control over growth conditions, compared to greenhouse production. However, producers are beginning to produce cannabis for the recreational market under greenhouse conditions, as it allows for larger cultivation areas and the use of natural sunlight, which reduces heating and lighting costs. To date, literature is scarce around best practices for cannabis growing methods. Several cultivation methods are used within growing rooms, including traditional bench setups, aeroponics, and hydroponics. While, Potter (2014) reviewed growing conditions used in industry they did not provide comparisons of productivity based on growing methods. While growers are keen to obtain high yields in each growth cycle, another challenge is the ability to obtain the maximum number of growing cycles per year (personal communication).

With the adoption of the Cannabis Act, Health Canada regulations will allow for outdoor cultivation of cannabis. While differences certainly will exist, producers interested in outdoor production of cannabis could adopt knowledge developed for agronomic practices (fertilization, seeding rate, harvest time, etc.) for cultivation of hemp (Atal, 1961; Mechtler et al., 2004; Amaducci et al., 2008; Cosentino et al., 2012; Faux et al., 2013; Finnan and Burke, 2013a,b; Faux and Bertin, 2014; Aubin et al., 2015, 2016; Razumova et al., 2016). However, it remains unclear how these conditions will influence medical/recreational cannabis quality aspects (flower yield, cannabinoid concentration), which are different from hemp quality variables (seed yield, fiber content). Thus, these factors will need to be investigated in the context of field cultivation of medical/recreational cannabis. The remainder of this section focuses on factors that affect cannabis yield and quality in the context of indoor, controlled environment production.

#### Potential Role for Plant-Growth Promoting Rhizobacteria in Cannabis Production

The role of the phytomicrobiome in regulating plant growth has received significant attention in the recent scientific literature and has been the basis for many crop-yield-enhancing technologies (e.g., Backer et al., 2018). Several studies have surveyed the diversity of bacterial and fungal endophytes in medical/recreational cannabis and hemp and have found that colonization depends on the cannabis genotype, the plant tissue sampled and the timing of sample collection relative to the plant growth stage. Among plants sampled from India, Pakistan, the USA and Canada the most common bacterial genera associated with medical/recreational cannabis and hemp plants were *Pseudomonas, Staphylococcus, Bacillus, Acinetobacter, Chryseobacterium, Enterobacter*, and *Microbacterium* while *Erwinia, Cedecia, Chryseobacterium, Enterobacter, Microbacterium* were found but at lower frequencies (Gautam et al., 2013; Winston et al., 2014; Afzal et al., 2015; Scott et al., 2018). These studies also determined that the colonization frequency was highest for leaves, followed by stems and petioles, however, these studies did not consider bacteria residing in or near root tissue. Community composition was determined mainly by soil type while community structure was determined by cultivar. These results highlight the need for systemic studies of microbial diversity in cannabis, with time points spanning from seed germination through to maturity, including leaf, stem, petiole, flower, and root tissue.

Many of the isolates identified in the studies mentioned above tested positively *in vitro* for properties associated with plant growth promotion (siderophore, cellulose, organic acid, and/or indole-3-acetic acid production and/or P-solubilization). *In planta*, two isolates were able to increase canola (*Brassica napus*) root length under salt stress conditions Afzal et al. (2015), while other isolates did not increase growth variables of tomato (*Solanum lycopersicum* L.) or hemp seedlings (Scott et al., 2018). Bioprospecting from wild cannabis may reveal PGPR that improve cannabis growth (Kusari et al., 2017). Alternatively, PGPR isolated from other crops may provide significant potential for improving cannabis yields. For example, Conant et al. (2017) reported that Mammoth P^TM^, a consortium of P-mobilizing microorganisms, increased flower yield per plant by 16.3% from 15.9 g (control) to 18.5 g (with Mammoth P^TM^). Since P-solubilization is only one of a set of mechanisms that microbes can use to promote plant growth, these results represent a promising starting point and suggest that testing microbes that increase plant yield by other mechanisms is warranted. Additionally, PGPR from the genus *Bacillus* have been shown to accelerate time to flowering for crops such as banana (*Musa acuminata* cv. “Berangan”), marigold (*Tagetes erecta* L.) and carnation (*Dianthus carophyllus* L.) (Mia et al., 2005; Flores et al., 2007; Kumar et al., 2014). Achieving flowering in a shorter timespan would reduce the time to harvest for each growth cycle to help growers attain a higher number of harvests per year.

In addition to increasing dry flower yield, inoculation with PGPR has the potential to increase cannabinoid yield via elicitation; this has been previously demonstrated for secondary metabolites in other plant species (previously reviewed by Gorelick and Bernstein, 2017). Several studies, cataloged in Table 1, have tested the role of biotic and abiotic elicitors on the effects of cannabinoid biosynthesis, revealing the sensitivity of this pathway to external signals. In contrast, studies conducted by Mansouri et al. (2009a,b, 2011, 2013 and 2016), Mansouri and Asrar (2012), Mansouri and Rohani (2014) and Mansouri and Salari (2014) demonstrated that abscisic acid, cycocel, ethephon, gibberellic acid, and mevinolin can all alter cannabinoid biosynthesis. While Flores-Sanchez et al. (2009) tested a large number of elicitors for effects on cannabis hairy root cell cultures, this did not induce cannabinoid biosynthesis. Testing the same elicitors in whole plants could lead to up- or down-regulation of cannabinoid biosynthesis. Furthermore, it has been demonstrated that bacteria isolated from one crop or plant species can stimulate growth and induce systemic resistance other crop species (Smith et al., 2015; Fan, 2017). Therefore, bacteria isolated from other crop species could be tested for effects on cannabinoid biosynthesis in cannabis plants.

PGPR also offer the potential to close the yield gap by reducing yield losses due to plant pathogens. PGPR can reduce yield losses by (1) inhibiting pathogen growth *in planta* or in soil via antagonism, (2) inducing systemic resistance in the plant, (3) reducing contamination between growth cycles. Strong evidence exists in the scientific literature to support the first mechanism. Endophytes isolated cannabis plants demonstrated their potential antagonistic activity against *Aspergillus flavus, Botrytis cinereal, Ceratocystis fimbriata, Colletotrichum gloeosporioides, Curvularia lunata, Fusarium oxysporum, Geotrichum candidum, Fusarium solani, Rhizoctonia solani, Sclerotinia sclerotiorum*, and *Trichothecium roseum, in vitro* (Gautam et al., 2013; Kusari et al., 2013; Qadri et al., 2013; Scott et al., 2018). The second option, inducing ISR, is of particular interest in cannabis production given (1) the high susceptibility of flowers to infection by plant pathogens and (2) the necessity to maintain extremely low pesticide residue levels on flowers. However, this remains to be tested in cannabis. Finally, harnessing the biocontrol aspect of PGPR to clean growing rooms in between growth cycles could reduce the risk of contamination between batches and reduce time between growth cycles.

Several studies have already investigated the role of endophytes in cannabis growth and development; while data are still lacking about effects on growth and yield of cannabis and the accumulation of cannabinoids in response to plant inoculation with PGPR. In contrast, multiple reports have investigated the role of cannabis endophytes for biocontrol; these have demonstrated strong potential for control of fungal and bacterial pathogens *in vitro*. The role of cannabis endophytes for biocontrol remains to be tested *in planta*.

## Conclusions

In order to increase cannabis yield per square meter and per W light, the results of this meta-analysis point to the use of (1) low plant density (≤12 plants per square meter), (2) a flowering period duration of 9 weeks, (3) the use of HPS lamps, (4) an adequate fertilizer regime, and (5) manipulating light intensity to preserve high energy efficiency vs. favor THC and CBD accumulation. Furthermore, our results demonstrate that cannabis varieties respond differently to production conditions. The vast amount of existing genomic and transcriptomic data can be used to catalog current cannabis diversity resulting from thousands of years of breeding and used to identify area for crop improvement. While these basic production conditions are further investigated, we also propose the use of additional technologies such as LEDs to increase power-use efficiency, and PGPR to increase nutrient efficiency and regulate cannabinoid yield.

## Author Contributions

RB, OW, and DS conceptualized the review layout. RB wrote and edited the review. PR, VM, SE, DL, and MA contributed significant portions of the text. TS conducted the meta-analysis with input from RB. RB and TS produced figures and tables. GR, ML, OW, and DS critically reviewed the manuscript.

### Conflict of Interest Statement

GR is Chief Executive Officer of Ravenquest Biomed, Inc., a company that produces and sells cannabis products; DS and OW conduct research in collaboration with this company, where the research is funded through the Natural Sciences and Engineering Research Council which levers industrial funding. The remaining authors declare that the research was conducted in the absence of any commercial or financial relationships that could be construed as a potential conflict of interest.

## Supplementary Material

Table S1 Projected and actual yield data for commercial cannabis operations in Canada as of April 2018. Actual yields are reported for AB Labs, United Greeneries, MedReleaf, Mettrum, WeedMD and Canopy Growth. Facility numbers corresponds to those shown in Figure 1 of the text. Data were collected from press releases provided by the companies, with links provided in column G.

Table S2 Yield data for cannabis (drug type) from the scientific literature based on growing conditions reported by authors. Units are as indicated in each column title. HPS, high pressure sodium; MH, metal halide; org, organic fertilizer (no further details provided by author); slowrel, slow release fertilizer contained in growing medium; Cap2017, Caplan et al., 2017; Con2017, Conant et al., 2017; Pot2012, Potter and Duncombe, 2012; Pot2014, Potter, 2014; Van2011, Vanhove et al., 2011; Van2012, Vanhove et al., 2012.

Table S3 Cannabis yield data included in the statistical analysis. Data for Caplan et al. (2017), Conant et al. (2017), and Potter and Duncombe (2012) were excluded from the meta-analysis due to a high degree of missing information about growing conditions. Units are as indicated in each column title. HPS, high pressure sodium; MH, metal halide; slowrel, slow-release fertilizer contained in growing medium; Pot2012, Potter and Duncombe, 2012; Van2011, Vanhove et al., 2011; Van2012, Vanhove et al., 2012.
