# Supplementary material for: Closing the Yield Gap for Cannabis: A Meta-Analysis of Factors Determining Cannabis Yield
Source: Front Plant Sci. 2019 Apr 24;10:495. doi: 10.3389/fpls.2019.00495 (PMC6491815; doi:10.3389/fpls.2019.00495)
Supplement: Supplementary file 1 [file Table_4.DOCX]

Based on the data studied, yield plant^-1^ can be predicted using the formula:

$$\ln\left( Yield {plant}^{-1} \right)=3.2138+\left( -.2501\times D \right)+\left( \begin{matrix} L_{int} & b_{2} \\ 270 & 0 \\ \begin{matrix} 400 \\ 600 \end{matrix} & \begin{matrix} 0.03523 \\ 0.3418 \end{matrix} \end{matrix} \right)+\left( \begin{matrix} L_{int} & V_{SH9} & b_{3} \\ 600 & 1 & 0.4402 \end{matrix} \right)$$

where *D* is plant density on the statistically standardized scale, L*_int_* is light intensity (W m^-2^) and *V_SH9_* = 1 indicates Silver Haze #9. Yield plant^-1^ increased with light intensity for varieties other than Silver Haze #9 (*p* = 0.0229); yield per plant was higher for Silver Haze #9 than other varieties at a light intensity of 600 W m^-2^ (*p* = 0.0009; Figure S1A). Yield per plant decreased at planting densities above 12 plants m^-2^ (*p* = 0.0065) (Figure S1B).


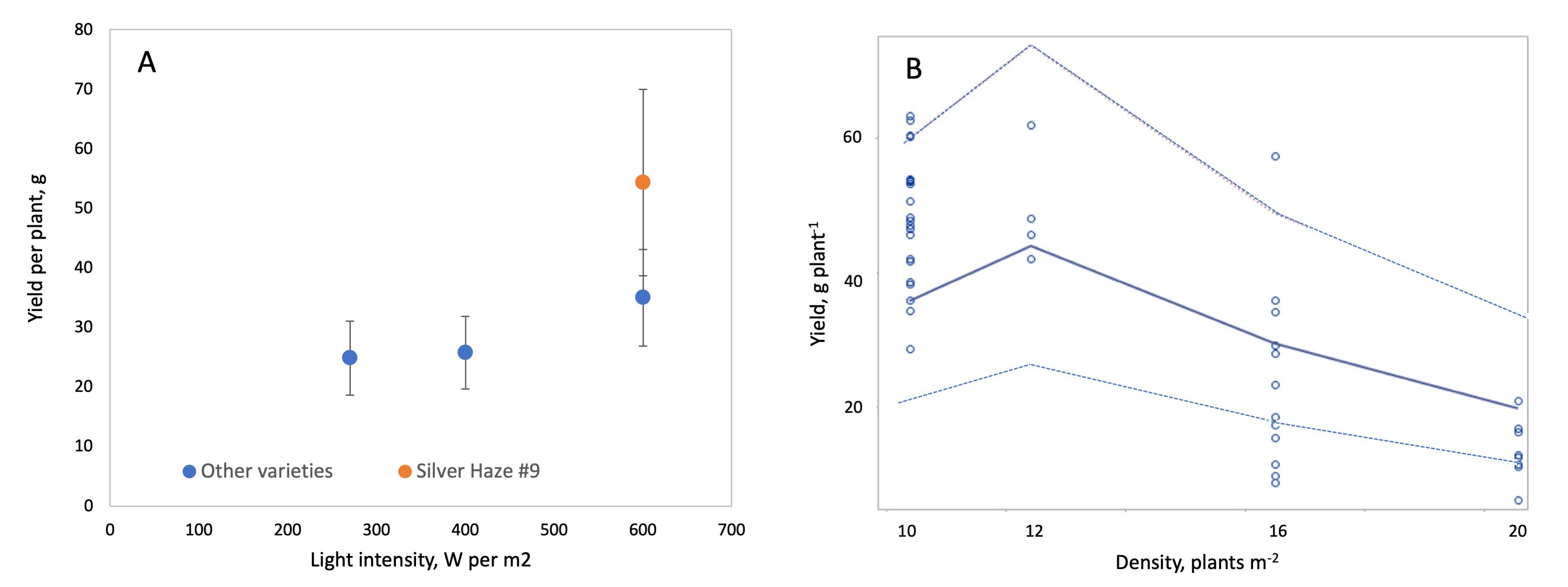


Figure S1.

THC plant^-1^ can be described according to:

$$ln\left( THC {plant}^{-1} \right)=8.5244+\left( 0.1505\times L_{int} \right)+\left( 0.2422\times P_{S}^{2} \right)+\left( F_{D}\times\left( \begin{matrix} V_{BB} & b_{3} \\ 1 & 0.8208 \\ 0 & 1.4194 \end{matrix} \right) \right)+\left( F_{D}\times\left( \begin{matrix} V_{SS} & b_{4} \\ 1 & -0.4766 \\ 0 & 0 \end{matrix} \right) \right)+\left( \begin{matrix} V_{EP} & F_{type} & b_{5} \\ 1 & 1 & -0.7776 \\ \begin{matrix} 0 \\ 0 \end{matrix} & \begin{matrix} 1 \\ 0 \end{matrix} & \begin{matrix} 0 \\ 0 \end{matrix} \end{matrix} \right)$$

where *L_int_* is light intensity on the statistically standardized scale, $P_{S}^{2}$ is the square of the pot size on the statistically standardized scale, *F_D_* is the duration of the flowering period on the statistically standardized scale, *V_BB_* = 1 represents Big Bud, *V_SS_* = 1 represents Super Skunk, *V_EP_* = 1 represents Early Pearly, and *F_type_* is fertilizer type (where 0 = CannaTerra and 1 = slow release fertilizer). THC plant^-1^ increased with light intensity and the square of the pot size (*p* = 0.0003 and *p* = 0.0159, respectively) (Figures S2A, B). Increasing the duration of the flowering period resulted in increased THC plant^-1^, but this effect was weaker for Big Bud and Super Skunk (Figures S2C, D) (*p* = 0.0003 and *p* = 0.0019, respectively). Early Pearly produced lower THC plant^-1^ compared other varieties when slow release fertilizer was applied (*p* < 0.0001).


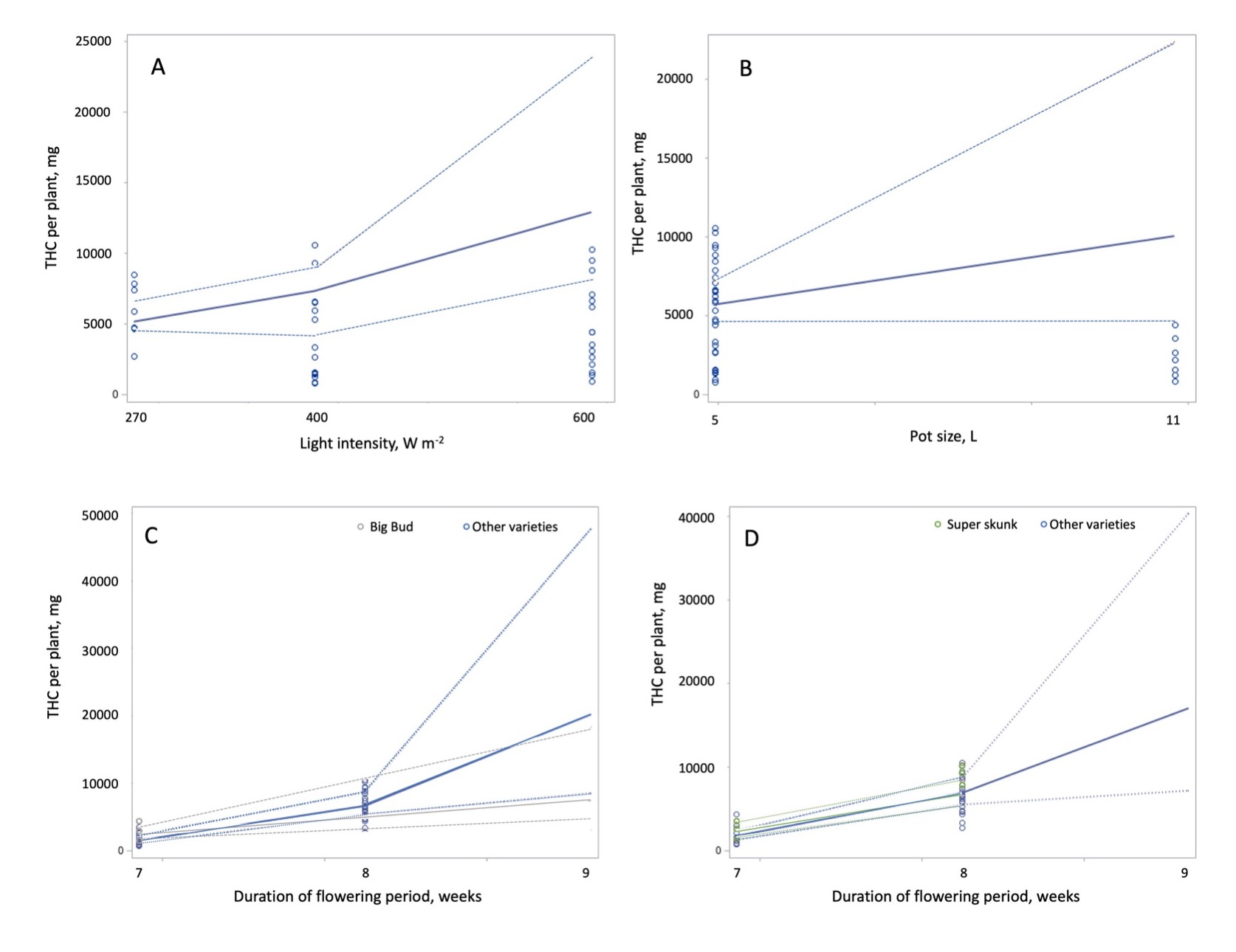
Figure S2.

CBD plant^-1^ can be described according to:

$$\frac{1}{\left( CBD {plant}^{-1} \right)^{2}}=0.000272+\left( \begin{matrix} L_{type} & L_{int} & b_{1} \\ 1 & 400 & 0.000755 \\ 0 & 600 & 0 \end{matrix} \right)$$

where *L_int_* is light intensity (W m^-2^) and *L_type_* is light type (where 0 = HPS and 1 = MH). When metal halide lamps with a light intensity of 400 W m^-2^ were used, CBD per plant was significantly lower than when high pressure sodium lamps with a light intensity of 600 W m^-2^ were used (*p* = 0. 0220) (Figure S3).


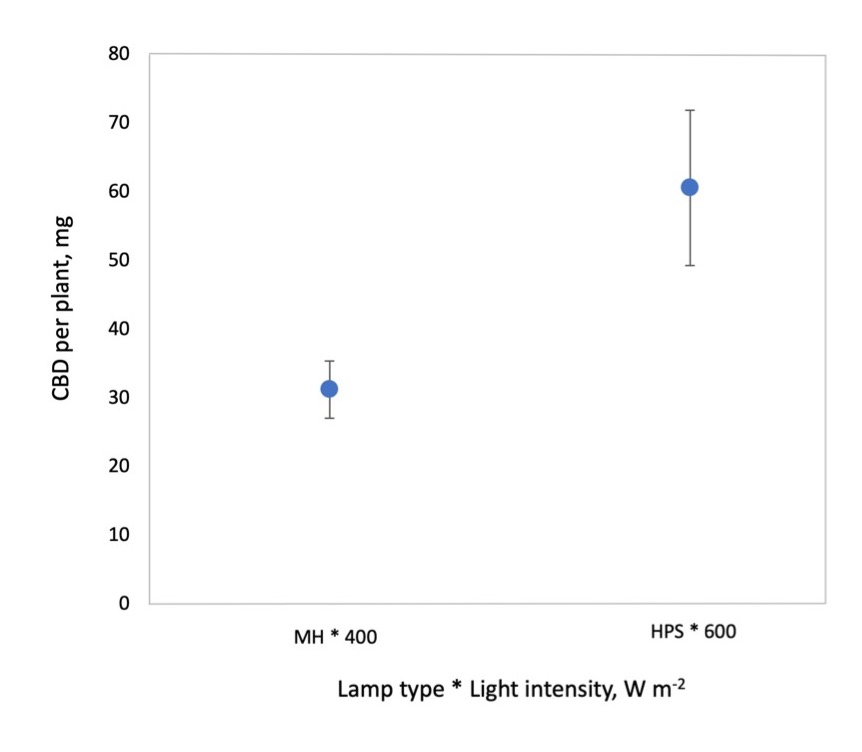


Figure S3.
